# Adiponectin Upregulates Prolyl-4-Hydroxylase α1 Expression in Interleukin 6-Stimulated Human Aortic Smooth Muscle Cells by Regulating ERK 1/2 and Sp1

**DOI:** 10.1371/journal.pone.0022819

**Published:** 2011-07-29

**Authors:** Li Li, Ke Zhang, Xiao-Jun Cai, Min Feng, Yun Zhang, Mei Zhang

**Affiliations:** 1 The Key Laboratory of Cardiovascular Remodeling and Function Research, Chinese Ministry of Education and Chinese Ministry of Public Health, Shandong University Qilu Hospital, Jinan, Shandong, China; 2 Jinan Central Hospital affiliated to Shandong University, Jinan, Shandong, China; Northwestern University, United States of America

## Abstract

Adiponectin is an anti-atherogenic adipokine that inhibits the development of plaque by mechanisms that are not completely understood. Extracellular matrix (ECM) may have a role in the pathogenesis of atherosclerosis. We explored the effect and mechanisms of adiponectin on the synthesis of prolyl-4-hydroxylase (P4H) in interleukin 6 (IL-6)-stimulated human aortic smooth muscle cells (HASMCs). P4Hα1 mRNA level was quantified by RT-PCR, the protein levels of phosphorylated extracellular signal-regulated kinase 1/2 (ERK1/2) and P4Hα1 were quantified by western blot analysis, and activation of specific protein 1 (Sp1) was determined by electrophoretic mobility shift assay and subcellular localization of Sp1 by immunofluorescence analysis. Adiponectin significantly increased P4Hα1 mRNA and protein levels in IL-6-stimulated HASMCs in a dose- and time-dependent manner. As well, ERK1/2 and Sp1 played a crucial role in the effect of adiponectin upregulating P4Hα1 expression in IL-6-stimulated HASMCs. Adiponectin abrogated the effects of IL-6 on collagen III level, which may indicate that P4Hα1 is essential for folding the procollagen polypeptide chains into stabilized collagen. Adiponectin attenuates IL-6–inhibited P4Hα1 synthesis and stabilizes collagen formation in HASMCs through a Sp1-ERK1/2-P4Hα1-dependent pathway.

## Introduction

Experimental results have demonstrated that the pathogenesis of atherosclerosis is based on several mechanisms. Extracellular matrix (ECM) may have a role in the pathogenesis of atherosclerosis [1]. ECM components, especially collagen, are thought to be important in the progression of atherosclerosis. Prolyl-4-hydroxylase (P4H) is a key intracellular enzyme essential for all known types of collagen maturation and secretion [2]. During collagen posttranslational processing, P4Hα1 folds the procollagen polypeptide chains into stable triple helical molecules [3]. Inhibition of P4H can decrease the level of collagen [4]. P4H is regulated by various cytokines, including tumor necrosis factor α, transforming growth factor β, and interleukins (ILs) [5]. Among them, IL-6 is one of the most potent cytokines involved in cardiovascular pathogenesis and actively regulates ECM metabolism [6]. Adiponectin is an adipocyte-specific plasma protein, has anti-inflammatory properties and might regulate ECM metabolism.

The promoter of P4Hα1 possesses several functional enhancer element-binding sites, including specific protein 1 (Sp1), NonO and hnRNP-K sites [7]. Sp1 is one of the first eukaryotic transcription factors to be identified and cloned [8]. It plays an important role in the transcriptional regulation of ECM metabolism. Previous work has shown that mutations in the Sp1-binding sites of the TbRE are associated with low collagen expression [9]. Although accumulating data have demonstrated that cytokines might decrease P4Hα1 expression through pathways leading to the activation of nuclear proteins, little is known about the defense molecules – responsive cis-elements – of the P4Hα1 transcriptional promoter.

Intracellular signaling transduction pathways activated by cytokines are mitogen-activated protein kinase (MAPK) pathways, of which there are 3 distinct groups: extracellular signal-regulated kinase (ERK), Jun N-terminal kinase, and p38 MAPK. ERK is most associated with the biological effect of inflammation, adiponectin and ECM metabolism. Some studies demonstrated that inflammation and adiponectin are involved in the ERK1/2 pathway. Other studies demonstrated that ECM metabolism is also involved in the pathway [10]. Sp1, as a vital downstream molecule in the MAPK pathway, can be activated by ERK to induce the DNA binding activity[11].

We aimed to investigate whether adiponectin can upregulate P4Hα1 expression in IL-6-stimulated human aortic smooth muscle cells (HASMCs) and to elucidate the corresponding mechanisms to find novel approaches to the management of cardiovascular risk.

## Materials and Methods

### Materials

HASMCs, cell culture medium and supplements were from Cell Science (Carlsbad, CA, USA). Recombinant human IL-6 was from R&D Systems (Minneapolis, MN, USA). Antibodies against P4Hα1 and Sp1 were from Abcom (Cambridge, MA, USA). Antibodies against phosphor-ERK1/2 and β-actin were from Santa Cruz Biotechnology (Santa Cruz, CA, USA). PD98059 was from Biosource (Camarillo, CA, USA). WP631 was from Alexis Biochemicals (San Diego, CA, USA). All other chemicals were of the highest grade commercially available.

### Cell Culture

HASMCs were cultured in smooth muscle cell culture medium up to passage 4. In all experiments, HASMCs were incubated in a humidified incubator at 37°C in a 95% air-5% CO_2_ atmosphere until cells reached 80%–90% confluence and were rendered quiescent by serum-free starvation for at least 24 h. HASMCs were stimulated with IL-6 in the presence or absence of 10 multiplicities of infection (MOI) of adiponectin adenovirus (Invitrogen, Carlsbad, CA, USA) for 8 hr. HASMCs were pretreated with 20 µM of the ERK inhibitor PD98059 or 0.1 µM of the Sp1 inhibitor WP631 for 1 hr.

### RT-PCR Analysis

After stimulation with 20 ng/ml IL-6 for 24 hr, RNA from cultured HASMCs was extracted with use of TriZol (Invitrogen, Carlsbad, CA, USA) following the manufacturer's protocol. Purified RNA was reverse-transcribed into cDNA (RevertAid M-MulV Reverse Transcriptase, Fermentas UAB, Mainz, Germany). Aliquots of the reverse-transcribed mixture underwent PCR with specific primers as described [12]. Results from quantitative real-time PCR were analyzed by use of the 7500 Real-Time PCR System (Applied Biosystems, Foster City, CA, USA). Results were controlled for real-time efficiency and normalized with those for β-actin.

### Western blot analysis

After stimulation with 20 ng/ml IL-6, cell lysates from cultured HASMCs were prepared with use of lysis buffer. Equal amounts of lysates were run on 14% poly-acrylamide gel, transferred onto nitrocellulose membranes (Bio-Rad, Hercules, CA, USA) and incubated at 4°C overnight with the primary antibodies mouse anti-β-actin (Santa Cruz Biotechnology; 1∶1000 dilution), goat anti-P4Hα1 (Abcom; 1∶250) or rabbit anti-phospho-specific p44/42 MAPK (Thr202 and Tyr204) (Santa Cruz Biotechnology; 1∶250). The blots were then incubated with peroxidase-conjugated secondary antibodies (Santa Cruz Biotechnology; 1∶2000 dilution) at room temperature for 2 hr, and membranes underwent detection by enhanced chemiluminescence plus reagents (Millipore, Billerica, MA, USA).

### Electrophoretic mobility shift assay (EMSA) of Sp1

Briefly, cultured HASMCs were pretreated with WP631 for 1 hr, then stimulated with 20 ng/ml IL-6 for 1 hr. Nuclear extracts were prepared (Active Motif, Carlsbad, CA, USA); the nuclear fraction was transferred into a pre-chilled microcentrifuge tube and stored at −80°C until use. The protein concentration of the cell extract was measured by the Bradford assay (Bio-Rad, Hercules, CA, USA). Activation of Sp1 was determined by EMSA (Roche, Madison, WI, USA) according to the manufacturer's instructions. The generated chemiluminescent signals were recorded on an imaging device.

### Immunofluorescence

Subcellular localization of Sp1 was determined by immunofluorescence analysis. Cultured HASMCs were pretreated with the Sp1 inhibitor WP631 or ERK inhibitor PD98059 for 1 hr before stimulation with 20 ng/ml IL-6 for 1 hr. Then cells were fixed with paraformaldehyde. Permeabilized cells were stained by direct immunofluoresence with a FITC-conjugated monoclonal antibody against Sp1 (1∶100 dilution). Inclusion-containing cells were visualized under a light fluorescent microscope (Olympus, Japan) and a Bio-Rad MRC 600 confocal laser scanning microscope (Bio-Rad, Hercules, CA).

### ELISA

Cultured HASMCs were pretreated with WP631 or PD98059 for 1 hr. After stimulation with 20 ng/ml IL-6 for 24 hr, the levels of type I and III procollagen and collagen secreted from HASMCs were quantified by ELISA (R&D Systems) according to the manufacturer's recommended protocols.

### Statistical Analysis

Data are presented as mean±SD. Statistical analyses involved ANOVA with subsequent Scheffe *t* test. A *P*<0.05 was considered statistically significant.

## Results

### 1. Effect of dose and time of IL-6 stimulation on P4Hα1 expression

**Figure 1:**
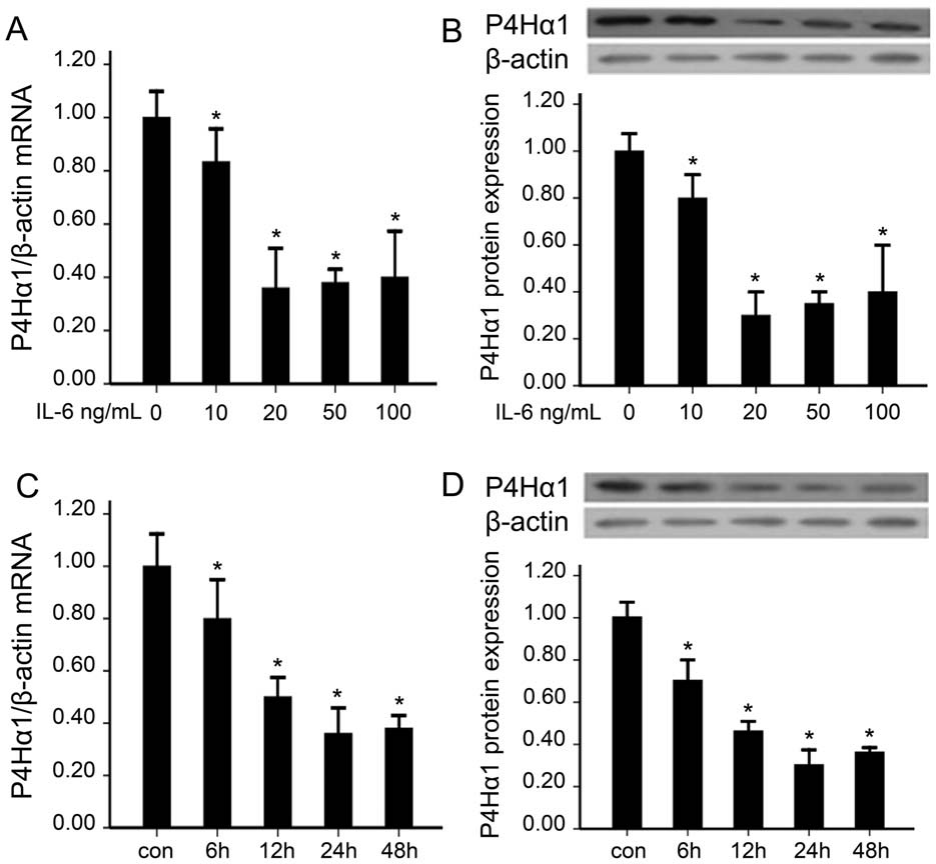
Effect of dose and time of IL-6 treatment on P4Hα1 expression. (A and C) mRNA expression of P4Hα1. (B and D) Protein expression of P4Hα1. Data are representative of 3 independent experiments (means±SD). *, *p*<0.05 vs. con.

To explore the effect of IL-6 on the expression of P4Hα1, HASMCs were treated with different doses of recombinant human IL-6 and for different times before being harvested for measurement of P4Hα1 mRNA and protein levels. The expression of P4Hα1 was lowest at 20 ng/ml IL-6 and at 24 hr (Figure 1).

### 2. Effect of dose and time of adiponectin on P4Hα1 expression

**Figure 2:**
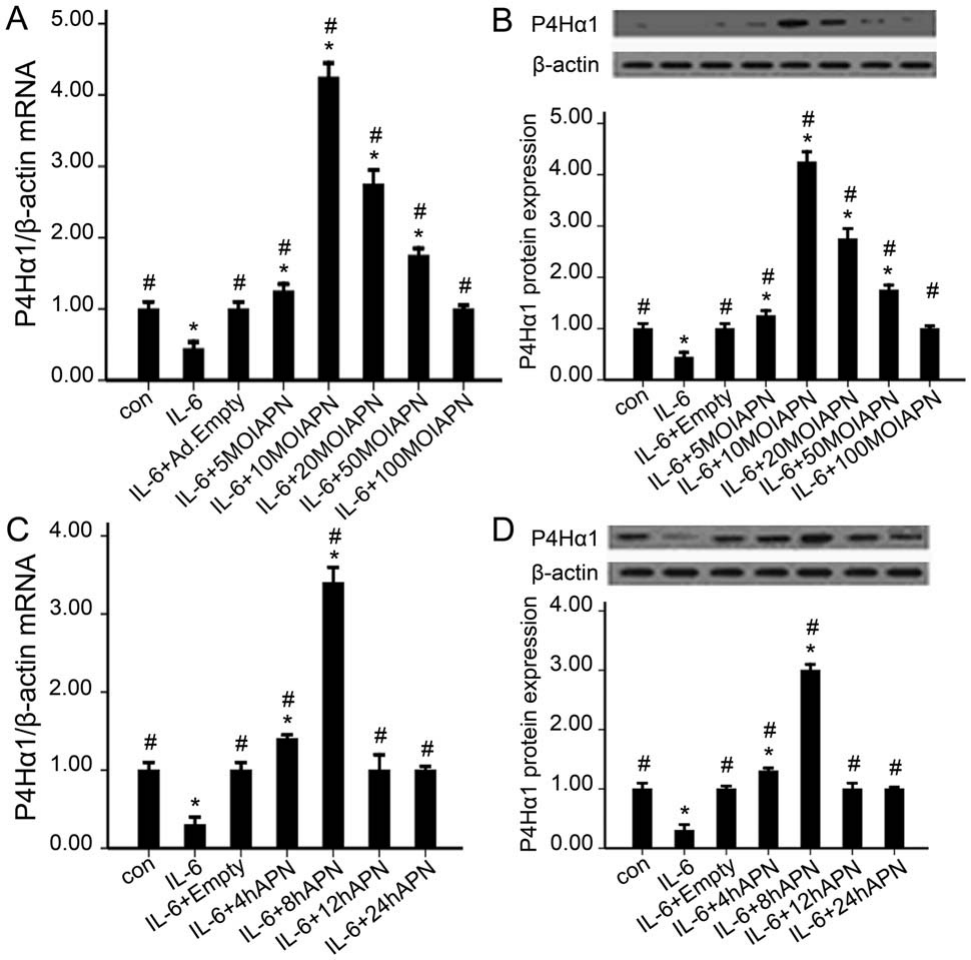
Effect of dose and time of adiponectin treatment on to P4Hα1 expression. (A and C) mRNA expression of P4Hα1. (B and D) Protein expression of P4Hα1. Data are representative of 3 independent experiments (means±SD). *, *p*<0.05 vs. con; #, *p*<0.05 vs. IL-6.

IL-6–stimulated HASMCs were treated with various MOI of adiponectin adenovirus for different times before being harvested for measurement of P4Hα1 mRNA and protein levels. The mRNA and protein expression of P4Hα1 peaked at 10 MOI adiponectin and at 8 hr (Figure 2).

### 3. Sp1 regulates the effect of adiponectin upregulating P4Hα1 expression in IL-6-stimulated HASMCs

#### 3.1 Effect of adiponectin on Sp1 activation induced by IL-6 in HASMCs

**Figure 3:**
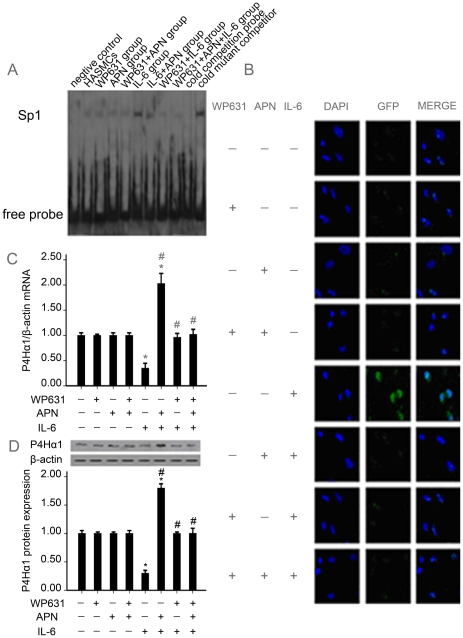
Sp1 regulates the effect of adiponectin upregulating P4Hα1 expression in IL-6-mediated HASMCs. (A) Sp1 DNA binding activity. (B) Subcellular localization of Sp1. (C) mRNA expression of P4Hα1. (D) Protein expression of P4Hα1. *, *p*<0.05 vs. con; #, *p*<0.05 vs. IL-6.

IL-6 significantly upregulated the binding activity of Sp1 in HASMCs as compared with control cells (*P*<0.05; Figure 3A). Adiponectin alone had no effect on the binding activity of the Sp1 in HASMCs, but it inhibited the IL-6-induced binding activity of Sp1 by 60% (*P*<0.05). Treatment with WP631, an inhibitor of Sp1, inhibited the IL-6 induced binding activity of Sp1 by 95% (*P*<0.05). The combination of WP631 and adiponectin abrogated the Sp1 binding activity induced by IL-6 by 90% (*P*<0.05), whereas adiponectin or WP631 alone did not affect Sp1 binding activity (*P*>0.05).

#### 3.2 Effect of Adiponectin on Subcellular Localization and Expression of Sp1 Induced by IL-6 in HASMCs

Most unstimulated cells showed diffuse staining in the cytoplasm, as well as weak staining in the nucleus, under normal growth conditions (Fig. 3B). With IL-6 treatment for 1 hr, staining for Sp1 in the nucleus was eight-fold higher than that in control cells (*P*<0.05). Eight hours after the addition of adiponectin to the cells, the level of nuclear staining was abrogated by 70% (*P*<0.05). After preincubation with WP631 for 1 hr, the level of nuclear staining was weak (*P*<0.05); the combination of WP631 and adiponectin also showed weak staining in the nucleus (*P*<0.05), which confirms the specificity of the Sp1 inhibitor used. However, staining for Sp1 in the nucleus was not influenced by adiponectin or WP631 alone (*P*>0.05).

#### 3.3 Effect of adiponectin on the expression of P4Hα1 Induced by IL-6 in HASMCs

IL-6 significantly downregulated the level of P4Hα1 mRNA by 65% (*P*<0.05) and protein by 70% (*P*<0.05) as compared with control cells (Fig. 3C,D). Eight hours after the addition of adiponectin to the cells, the level of P4Hα1 mRNA was increased 5.8-fold (*P*<0.05) and that of P4Hα1 protein six-fold (*P*<0.05). After preincubation with WP631 for 1 hr or the Sp1 inhibitor WP631 and adiponectin combined, the level of P4Hα1 mRNA and protein returned to the level of control cells. However, the expression of P4Hα1 was not influenced by adiponectin or WP631 alone (*P*>0.05).

### 4. ERK1/2 leads to adiponectin upregulating P4Hα1 expression in IL-6- mediated HASMCs in an Sp1-dependent manner

#### 4.1 Effect of adiponectin on phosphorylation of ERK induced by IL-6 in HASMCs

**Figure 4:**
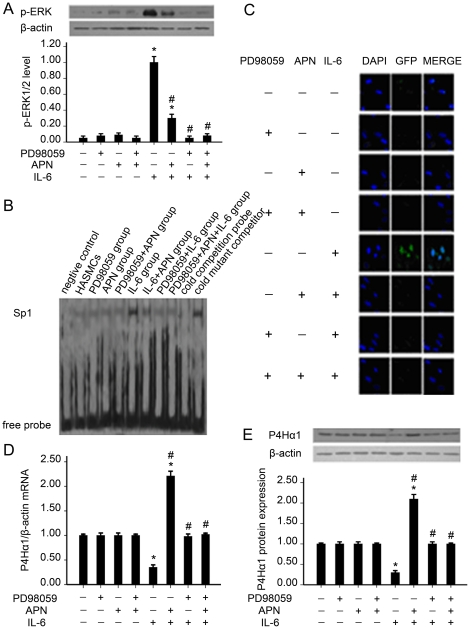
ERK1/2 leads to adiponectin upregulating P4Hα1 expression in IL-6-stimulated HASMCs. (A) p-ERK level. (B) Sp1 DNA-binding activity. (C) Subcellular localization of Sp1. (D) mRNA expression of P4Hα1. (E) Protein expression of P4Hα1. *, *p*<0.05 vs. con; #, *p*<0.05 vs. IL-6.

IL-6 significantly induced the phosphorylation of ERK in HASMCs as compared with control cells (*P*<0.05) (Fig. 4A). Eight hours after the addition of adiponectin to the cells, the phosphorylation of ERK induced by IL-6 was abrogated by 70% (*P*<0.05). After preincubation with PD98059 for 1 hr, the phosphorylation of ERK induced by IL-6 was abrogated by 95% (*P*<0.05). After preincubation with PD98059 and adiponectin combined, the phosphorylation of ERK induced by IL-6 was abrogated by 92% (*P*<0.05). However, the phosphorylation of ERK was not influenced by adiponectin or PD98059 alone (*P*>0.05).

#### 4.2 Effect of ERK on Sp1 activation induced by IL-6 in HASMCs

IL-6 significantly upregulated the binding activity of Sp1 in HASMCs as compared with control cells (*P*<0.05) (Fig. 4B). Eight hours after the addition of adiponectin to the cells, the binding activity of Sp1 induced by IL-6 was reduced by 70% (*P*<0.05). After preincubation with PD98059 for 1 hr, the binding activity of Sp1 induced by IL-6 was reduced by 95% (*P*<0.05). The combination of PD98059 and adiponectin reduced the IL-6-induced binding activity of Sp1 by 93% (*P*<0.05). However, the binding activity of Sp1 was not influenced by adiponectin or PD98059 alone (*P*>0.05).

#### 4.3 Effect of ERK on subcellular localization and expression of Sp1 induced by IL-6 in HASMCs

Most unstimulated cells had diffuse staining in the cytoplasm, as well as weak staining in the nucleus, under normal growth conditions (Fig. 4C). With 1-h treatment with IL-6, staining for Sp1 in the nucleus was increased seven-fold in HASMCs as compared with control cells (*P*<0.05). Eight hours after the addition of adiponectin, the level of nuclear staining was reduced by 70% (*P*<0.05). After preincubation with PD98059 for 1 hr, the level of nuclear staining was weak (*P*<0.05). The combination of PD98059 and adiponectin also produced weak staining in the nucleus (*P*<0.05). However, staining for Sp1 in the nucleus was not influenced by adiponectin or PD98059 alone (*P*>0.05).

#### 4.4 Effect of ERK on the expression of P4Hα1 induced by IL-6 in HASMCs

IL-6 significantly downregulated the level of P4Hα1 mRNA by 65% (*P*<0.05) and protein by 70% (*P*<0.05) in HASMCs as compared with control cells (Fig. 4D,E). Eight hours after the addition of adiponectin, the level of P4Hα1 mRNA was increased 6.3-fold (*P*<0.05) and protein seven-fold (*P*<0.05). After preincubation with PD98059 for 1 hr or the combination of PD98059 and adiponectin, the level of P4Hα1 mRNA and protein returned to the level of control cells (*P*<0.05). However, the expression of P4Hα1 was not influenced by adiponectin or PD98059 alone (*P*>0.05).

### 5. Effect of adiponectin overexpression on collagen production

**Figure 5:**
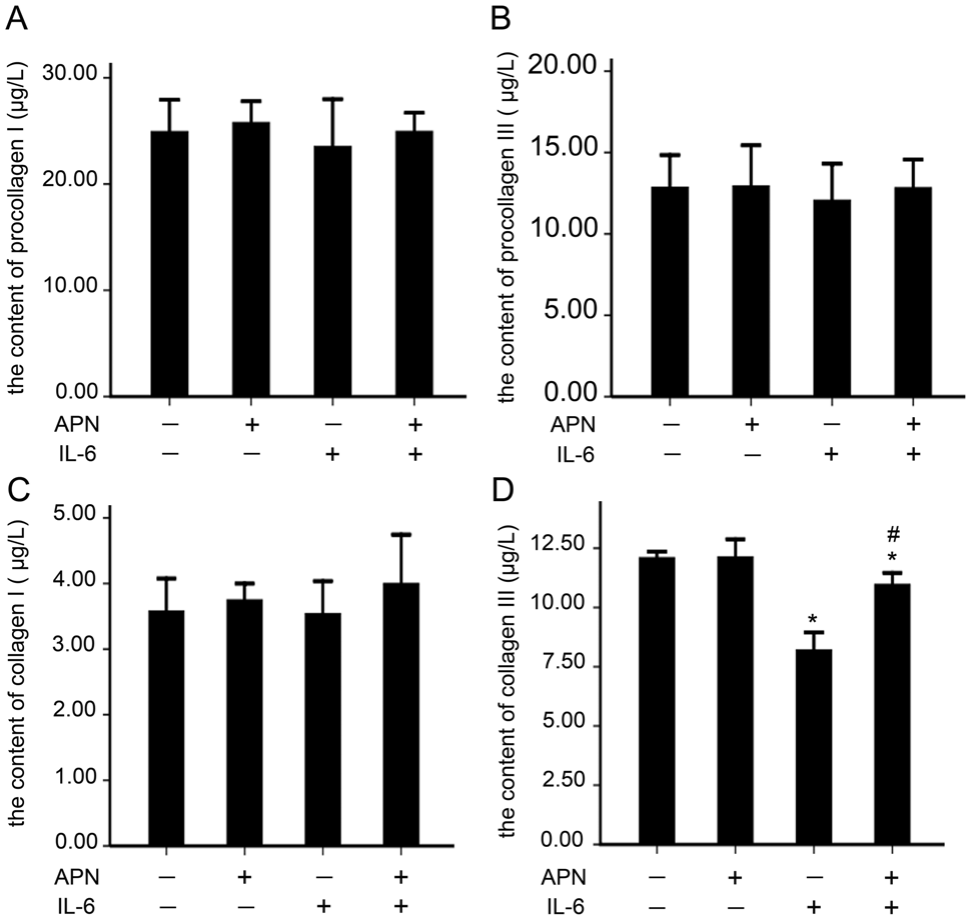
Effect of adiponectin overexpression on collagen production. Content of (A) procollagen I, (B) procollagen III, (C) collagen I, (D) collagen III. Data are representative of 3 independent experiments (means±SD). *, *p*<0.05 vs. con; #, *p*<0.05 vs. IL-6.

The content of procollagen I and III did not change significantly with or without adiponectin or IL-6 treatment (Fig. 5A,B), whereas after the addition of IL-6 to the cells, the content of collagen III decreased significantly in HASMCs as compared with control cells (IL-6: 8.20±0.3 µg/L vs. control: 12.1±0.1 µg/L; *P*<0.05) (Fig. 5D). Eight hours after the addition of adiponectin to the cells, the content of collagen III increased significantly (IL-6: 8.20±0.3 µg/L vs. IL-6+APN: 10.97±0.2 µg/L; *P*<0.05) (Fig. 5D), whereas the content of collagen I did not change significantly in all groups (Fig. 5C).

## Discussion

Arterial interstitial collagen confers tensile strength on the fibrous cap and thus determines plaque stability and vulnerability to rupture. Therefore, the synthesis of collagen in the fibrous cap may be directly responsible for plaque rupture[13]. In this study, we aimed to investigate the role of ECM in the pathogenesis of atherosclerosis and the potential mechanism of adiponectin upregulating P4Hα expression in IL-6-stimulated HASMCs. First, we determined that adiponectin can upregulate the expression of P4Hα1 in HASMCs induced by IL-6. Second, we showed that adiponectin upregulating P4Hα1 expression in IL-6-mediated HASMCs depended in part on ERK1/2 activation and Sp1 DNA binding activity. Adiponectin also increased collagen III level in HASMCs alone with upregulated P4Hα1 expression.

Adiponectin is an adipose tissue-secreted protein with anti-inflammatory and anti-atherogenic properties. A proinflammatory state in adipose tissue can reduce secretion of adiponectin and increase that of several cytokines, including TNF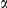, IL-6, and other proinflammatory molecules [14]. Phosphorylation of GATA2 by Akt increases adipose tissue differentiation and reduces adipose tissue–related inflammation [15]. Other studies have investigated the ratio of TNF to adiponectin and the consequences for inflammation and atherosclerosis. Atherosclerosis was ameliorated in gAd Tg apoE-deficient mice, which was associated with decreased expression of class A scavenger receptor and TNF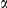 [16]. Full-length adiponectin was found to upregulate TNF-α and IL-6 protein production differently [17]. In our research, adiponectin attenuated the IL-6–inhibited P4Hα1 synthesis in HASMCs and consequently changed the content of collagen. All of these data suggest the significant inverse relationship between level of adiponectin and IL-6 or TNF-α associated with the development of inflammation-related atherosclerosis. Although adiponectin has anti-inflammatory and antiatherogenic properties, it has no effect on normal cells, unless in combination with inflammation. Adiponectin significantly suppressed LPS-induced IL-6 and monocyte chemoattractant protein 1 production and also significantly reduced NF-kappaB activity and IkappaB-alpha and IKK gene expression as compared with 3T3-L1 induced by LPS alone [18]. Adiponectin may reduce LPS-induced NO production and nitrosative stress and prevent adventitial fibroblasts (AFs) from proliferating, transforming to myoflbroblasts, and migrating to the intima, thus worsening atherosclerosis, by inhibiting the AdipoR1-AMPK-iNOS pathway in AFs [19]. Our present results also show that adiponectin had no effect on P4Ha1 expression, Sp1 DNA binding activity or ERK phosphorylation under basal conditions. With IL-6 stimulation, adiponectin reverted the IL-6-induced abnormal P4Ha1 expression, Sp1 DNA binding activity and ERK phosphorylation to the normal level at least 2 times more than the control. Therefore, adiponectin has anti-inflammatory properties and might regulate inflammatory responses in atherosclerotic lesions.

Several studies have identified that adiponectin might regulate ECM metabolism in atherosclerotic lesions [20]-[22]. Our previous study of an atherolerosis mouse model indicates that adiponectin could significantly increase the expression of P4Hα1 in plaque, which may play a major role in the development of the thick fibrous cap of atherosclerotic plaque and prevent plaque rupture [23]. These data suggest that adiponectin might have anti-atherosclerotic properties and regulate ECM metabolism in atherosclerotic lesions, but the potential mechanism was unknown. We further studied the regulation of adiponectin on P4Hα1 expression *in vitro*. The expression of P4Hα1 was mostly induced by IL-6. Adiponectin could upregulate the expression of P4Hα1 in HASMCs induced by IL-6 in a dose- and time-dependent manner, so adiponectin might have anti-inflammatory properties and might regulate ECM metabolism.

To identify signaling pathways in this induction, we studied the activation of Sp1, a known transcriptional factor, that might play an important role in the transcriptional regulation of ECM metabolism [24]–[26]. IL-6, sIL-6R, or both were found to decrease both the ratio of Sp1 to Sp3 and DNA binding activities, thus inhibiting COL2A1 transcription [27]. Our results showed that Sp1 DNA binding activity was enhanced on exposure to IL-6. Both the mRNA and protein levels of P4Hα1 were significantly attenuated. To clarify that the effect depended on Sp1, we used the Sp1 inhbitor WP631, which efficiently inhibited Sp1 DNA binding activity. In the presence of the inhibitor, P4Hα1 mRNA and protein levels were significantly enhanced. Adiponectin could counteract all of these effects. Therefore, Sp1 contributed to the adiponectin-induced P4Hα1 expression.

MAPK pathways are important mediators of signal transduction and are involved in multiple intracellular signaling cascades and activated by cytokines [28]–[31]. Some studies demonstrated that inflammation, adiponectin and ECM metabolism are all involved in the ERK1/2 pathway [32]–[34]. Sp1, as a vital downstream molecule in the MAPK pathway, can be activated by ERK and induce the Sp1 DNA binding activity [35]. Our results showed phosphorylation of ERK1/2 and Sp1 DNA binding activity enhanced after exposure to IL-6. Both P4Hα1 mRNA and protein levels were significantly attenuated. To clarify that the effect depends on ERK1/2, we used the ERK1/2 inhbitor PD98059, which efficiently inhibited phosphorylation of ERK1/2 and Sp1 DNA binding activity. In the presence of the inhibitor, P4Hα1 mRNA and protein levels were significantly enhanced. Adiponectin could counteract all of these effects. These results supported that adiponectin upregulating P4Hα1 expression in IL-6-mediated HASMCs depended in part on ERK1/2 activation.

The organic matrix of a plaque mainly consists of types I and III collagen. Many studies have fully discussed their content and changes in content in atherosclerotic plaques. The completely processed and cross-linked type III collagen seems to be the major collagen type in atherosclerotic plaques [36]. The density of type III was increased in diabetes mellitus (DM) plaques with a non-significant reduction in type I density in DM as compared with non-DM plaques [37]. The pattern of GPVI-Fc binding was similar to the immunostaining pattern of collagen type III but differed from that of collagen type I, which was more intense in the cap than in the core [38]. We further explored the effect of adiponectin overexpression on collagen content and found no difference in the content of procollagen I and III on IL-6 stimulation with or without adiponectin treatment; only collagen III content was reversed by adiponectin. Therefore, type III collagen seem to be the major collagen type in atherosclerotic plaques. P4Hα1 is essential for collagen maturation and secretion by folding the procollagen polypeptide chains into stable triple helical molecules, so the change in collagen III content may reflect the activity of P4Hα1 in HASMCs.

In conclusion, we show for the first time that adiponectin attenuates the IL-6–inhibited P4Hα1 synthesis in HASMCs and therefore collagen III content was changed. This effect of adiponectin is through ERK1/2-dependent pathways and by inhibiting Sp1 activation. Our results suggest that adiponectin may play a major role in the development of the thick fibrous cap of atherosclerotic plaque and stabilize plaque by increasing P4Hα1 synthesis in HASMCs stimulated by IL-6.
